# Diamond‐like carbon coating enhances mechanical performance of NiTi rotary instruments: A multimethod analysis

**DOI:** 10.1111/iej.14253

**Published:** 2025-05-14

**Authors:** Emmanuel J. N. L. Silva, Tereza Vitória Mauri Lorenzoni, Mylena do Rosário Pereira, Victor T. L. Vieira, Jorge N. R. Martins, Francisco Manuel Braz Ferndandes, Marco A. Versiani

**Affiliations:** ^1^ School of Dentistry Grande Rio University (UNIGRANRIO) Rio de Janeiro Brazil; ^2^ Department of Endodontics Rio de Janeiro State University Rio de Janeiro Brazil; ^3^ Deparment of Endodontics Fluminense Federal University Niterói Brazil; ^4^ Faculdade de Medicina Dentária Universidade de Lisboa Lisbon Portugal; ^5^ Grupo de Investigação Em Bioquimica e Biologia Oral, Unidade de Investigação Em Ciências Orais e Biomédicas (UICOB), Faculdade de Medicina Dentária Universidade de Lisboa Lisbon Portugal; ^6^ Centro de Estudo de Medicina Dentária Baseada na Evidência (CEMDBE) ‐ Cochrane Portugal, Faculdade de Medicina Dentária Universidade de Lisboa Lisbon Portugal; ^7^ Laboratory LIBPhys ‐ FCT UID/FIS/04559/2013 Faculdade de Medicina Dentária, Universidade de Lisboa Lisbon Portugal; ^8^ CENIMAT/I3N, Department of Materials Science NOVA School of Science and Technology, Universidade NOVA de Lisboa Caparica Portugal; ^9^ Dental Specialty Center, Brazilian Military Police Belo Horizonte Brazil

**Keywords:** diamond‐like carbon coating, endodontics, mechanical properties, nickel–titanium instruments

## Abstract

**Aim:**

This study aimed to evaluate the impact of diamond‐like carbon (DLC) surface treatment on the mechanical properties of nickel–titanium (NiTi) rotary instruments.

**Methodology:**

One hundred and ten nickel–titanium instruments with a size of 25/.06 and a length of 25 mm, both with (*n* = 55) and without (*n* = 55) a DLC coating were selected and compared regarding their design (stereomicroscopy, scanning electron microscopy), metallurgy (energy‐dispersive X‐ray spectroscopy, differential scanning calorimetry), and mechanical performance (time to fracture, bending strength, buckling strength, cutting efficiency, and microhardness). Data were analysed using Mann–Whitney and independent Student's *t*‐test (α = 5%).

**Results:**

The design analysis confirmed that both instruments had identical geometric features, similar spiral and tip designs, with DLC‐coated instruments showing fewer surface irregularities and a multi‐coloured appearance. Metallurgical analysis revealed identical transformation temperatures for both groups, with the R‐phase starting at ~32°C, finishing at ~25°C during cooling, and the austenitic finish occurring at ~35°C. DLC‐coated instruments demonstrated significantly superior cyclic fatigue resistance (*p* = 0.0028), lower bending load (*p* = 0.0294), lower cutting efficiency (*p* < 0.0001), and higher microhardness (*p* = 0.0019), whilst no difference was observed in terms of buckling strength (*p* = 0.3569).

**Conclusions:**

Diamond‐like carbon surface treatment significantly enhanced cyclic fatigue resistance, flexibility, and microhardness of NiTi rotary instruments without compromising their structural integrity.

## INTRODUCTION

The development of nickel–titanium (NiTi) rotary instruments has revolutionized endodontics by enhancing the efficiency and predictability of root canal shaping whilst reducing errors (Bürklein & Arias, 2022). Advances in metallurgy and manufacturing have resulted in diverse NiTi instruments with variations in heat treatment, design, and kinematics to improve mechanical performance and clinical reliability (Martins et al., 2022; Silva et al., 2020). However, NiTi instruments remain susceptible to mechanical failure, especially in curved or calcified canals, where cyclic fatigue and torsional stress heighten the risk of fracture. This, in turn, can hinder canal debridement, disinfection, and overall clinical outcomes (McGuigan et al., 2013; Ng et al., 2011). To enhance the durability of NiTi instruments, heat treatment has emerged as a key innovation. By altering the alloy's crystallographic structure, this process increases flexibility, improves resistance to cyclic fatigue, and enhances adaptability to complex canal anatomies through a martensitic phase transformation (Zupanc et al., 2018). The growing adoption of heat‐treated NiTi instruments reflects their clinical benefits and the increasing focus on refining metallurgical processes to optimize performance.

In the last years, surface modifications have emerged as an additional strategy to further enhance the mechanical properties of NiTi instruments. Amongst the latest advancements in this area is the application of diamond‐like carbon (DLC) coatings, which involve the deposition of a thin, durable carbon film onto the instrument's surface through a solid‐state ionization process in a vacuum furnace. It has been demonstrated that DLC coatings offer significant mechanical benefits when applied to dental instruments (Silva, Crozeta, et al., 2023), enhancing durability and performance. These coatings are distinguished by their high hardness, which significantly improves wear resistance, particularly for instruments exposed to repeated mechanical stress and abrasion during clinical procedures (Hinüber et al., 2010; Malisz et al., 2023). The hardness of DLC coatings helps extend the lifespan of tools, reducing the frequency of replacement and ensuring long‐term reliability. Furthermore, the low coefficient of friction of DLC coatings minimizes wear, promoting smoother and more efficient movements of dental instruments, crucial for tasks requiring precision (Huang et al., 2013; Muguruma et al., 2013). Additionally, DLC coatings provide excellent corrosion resistance, shielding instruments from the harsh, often acidic conditions within the oral cavity and preventing metal degradation (Huang et al., 2013; Malisz et al., 2023). Collectively, these properties make DLC coatings particularly beneficial for endodontic instruments that are subjected to repetitive mechanical forces and require precise performance.

The recently launched RCS Rainbow NiTi system (Ramo Medical, Suzhou, China) is characterized by an S‐shaped cross‐section, proprietary heat treatment, and a distinctive multi‐coloured surface. According to the manufacturer, the colour variations result from a diamond‐like carbon (DLC) treatment (https://bit.ly/3HIqRvZ). A recent study compared RCS Rainbow instruments with different heat‐treated systems regarding their design, metallurgy, and mechanical properties, such as cyclic fatigue, torsional resistance, bending and buckling resistance, and cutting ability (Silva et al., 2024). The results suggest a well‐balanced set of properties in the instrument; however, since this study analysed multiple instruments with varying designs and metallurgical treatments, the specific contribution of surface modifications to these differences remains uncertain. Notwithstanding this promising finding, the true impact of DLC coatings on the overall mechanical behaviour and clinical performance of NiTi instruments remains largely unexplored. Understanding whether DLC coatings can enhance mechanical resistance and structural integrity without compromising the instrument's flexibility is crucial for determining their clinical applicability. To accurately assess the true impact of DLC treatment, it is important to isolate this variable from other factors that may influence the instrument's performance. This allows for a clear evaluation of DLC's specific role in the mechanical behaviour of NiTi instruments. Therefore, the aim of this study was to evaluate the effect of DLC surface treatment on the mechanical properties of rotary NiTi instruments. Two versions of the same instrument—identical in geometry and subjected to the same heat treatment—were tested. The only difference between them was the application of a DLC coating, which was present on one version and absent on the other. The null hypothesis stated that DLC surface treatment would not significantly affect the mechanical properties of the tested instruments.

## MATERIAL AND METHODS

This manuscript complies with the Preferred Reporting Items for Laboratory studies in Endodontology (PRILE) guidelines (Figure S1) and the respective checklist set forward by the International Endodontic Journal.

### Sample selection

One hundred and ten nickel–titanium instruments with a size of 25/.06 and a length of 25 mm, both with (*n* = 55) and without (*n* = 55) a DLC coating (Rainbow; Ramo Medical, Suzhou, China), were selected for the study. Prior to testing, all instruments underwent a meticulous microscopic inspection at 13.6× magnification under LED illumination (Opmi Pico; Carl Zeiss Surgical) to assess potential manufacturing defects. Specific attention was given to identifying irregularities in the cutting blades or signs of unwinding that could compromise their integrity. No defects were observed, and all instruments met the inclusion criteria.

### Design

Six randomly selected instruments from each group (with or without DLC coating) were examined under a dental microscope (Opmi Pico, Carl Zeiss Surgical, Jena, Germany) at 13.6× magnification, with high‐resolution images captured (Canon EOS 500D; Canon, Tokyo, Japan) to assess the active cutting blade length, number and direction of spirals, spiral density (spirals per millimetre), and potential manufacturing inconsistencies. Then, the instruments were secured in a file holder and analysed using a scanning electron microscope (SEM) (S‐2400, Hitachi, Tokyo, Japan) to evaluate spiral geometry, tip configuration, surface finishing marks, and potential structural irregularities.

### Metallurgy

Three instruments from each group were analysed using a conventional SEM (DSM‐962, Carl Zeiss Microscopy GmbH, Jena, Germany) with an Inca X‐act EDS detector (Oxford Instruments NanoAnalysis, Abingdon, UK) at 20 kV, 3.1 amps, and a 25 mm working distance. After a 10‐minute vacuum process, data acquisition occurred over 60 seconds, maintaining ~30% dead time and covering a 500 μm × 500 μm area from the blade's middle section. The semi‐quantitative EDS analysis, with ZAF correction (Microanalysis Suite v.4.14, Oxford Instruments), determined nickel and titanium proportions and detected other metallic elements. For DSC evaluations (DSC 204 F1 Phoenix; Netzsch‐Gerätebau GmbH, Selb, Germany), performed according to ASTM guidelines (ASTM International, 2004), three 4–5 mm fragments (5–10 mg) were collected from the active blades of both DLC and non‐DLC instruments at corresponding tip, middle, and coronal sections. Fragments underwent chemical etching with a solution of 25% hydrofluoric acid, 45% nitric acid, and 30% distilled water for 2 min, followed by neutralization. Positioned in an aluminium pan with an empty control pan, samples underwent a 1 h 40 min thermal cycle under nitrogen gas, including stabilization at ambient temperature, heating to 150°C (10°C/min), a 2‐minute plateau, cooling to −150°C (10°C/min), a second heating phase to 150°C, and final stabilization. Data were processed using Netzsch Proteus Thermal Analysis software (Netzsch‐Gerätebau GmbH).

### Mechanical performance

Five mechanical parameters were evaluated: time to fracture, bending strength, buckling strength, cutting efficiency, and microhardness. Sample size was determined using initial tests (*n* = 5), considering a 0.05 significance level, 80% statistical power, and effect sizes (mean ± SD): 27.2 ± 16.6 (fracture time), 63.2 ± 46.8 (bending strength), 0.6 ± 23.4 (buckling strength), 23.8 ± 13.2 (cutting efficiency), and 42.8 ± 30.2 (microhardness). The required sample sizes were 7, 10, 23877, 6, and 9, respectively. As buckling strength required an impractically large sample without meaningful clinical implications, it was excluded from the sample size calculation, and 10 instruments were selected based on the remaining parameters. For microhardness, 15 samples were used to accommodate five indentations per instrument.

The cyclic fatigue test was conducted using a 6:1 reduction handpiece (Sirona Dental Systems GmbH, Bensheim, Germany) powered at 400 rpm and 2.0 N torque (VDW Silver; VDW GmbH). Instruments were statically operated in a stainless‐steel curved tube (6 mm radius, 86° angle), with fracture time recorded via a digital chronometer and fragment size measured with a digital calliper (Mitutoyo, Aurora, USA). Bending resistance (gf) followed international standards (ISO 3630‐3631, 2008), whilst buckling load (N) was measured using a universal testing machine (1 kN load cell; Instron Corporation 4502; series H3307) (Lopes et al., 2012). Cutting efficiency was assessed using a custom apparatus linking an endodontic motor (VDW Gold) to a 500 N load cell (Silva, Ajuz, et al., 2023). Each instrument was positioned at the uppermost part of a simulated straight canal (size 15/.02) within a bone block model (PCF 10; Sawbones, Vashon, WA, USA), pre‐loaded with 10 gf by the universal testing machine, and then operated at 400 rpm and 2.0 N torque, advancing 3 mm forward and 2 mm backward per cycle, resulting in a net progression of 1 mm per cycle. Maximum force recorded during the test indicated cutting efficiency, with higher force implying lower cutting ability. Instrument microhardness was evaluated using a Vickers hardness tester (Duramin; Struers Inc., Cleveland, OH) following ASTM E384 standards (ASTM International, 2017), with specimens fixed onto acrylic blocks. Fifteen indentations were made—five per instrument in each group—using a diamond indenter with a 100 gf load for 15 seconds (De‐Deus et al., 2017). Hardness values, recorded as Hardness Vickers Number (HVN), were measured under ×40 magnification.

### Statistical analysis

Data normality was assessed using the Shapiro–Wilk test. Considering the data distribution, the results were reported as mean values with standard deviations or median values along with the first and third quartiles. The time to fracture, bending resistance, buckling resistance, and cutting ability were compared using the independent Student's *t*‐test, whilst microhardness was analysed using Mann–Whitney test. A significance level of 5% was set for all statistical comparisons (SPSS v22.0 for Windows; SPSS Inc.).

## RESULTS

### Design

**FIGURE 1:**
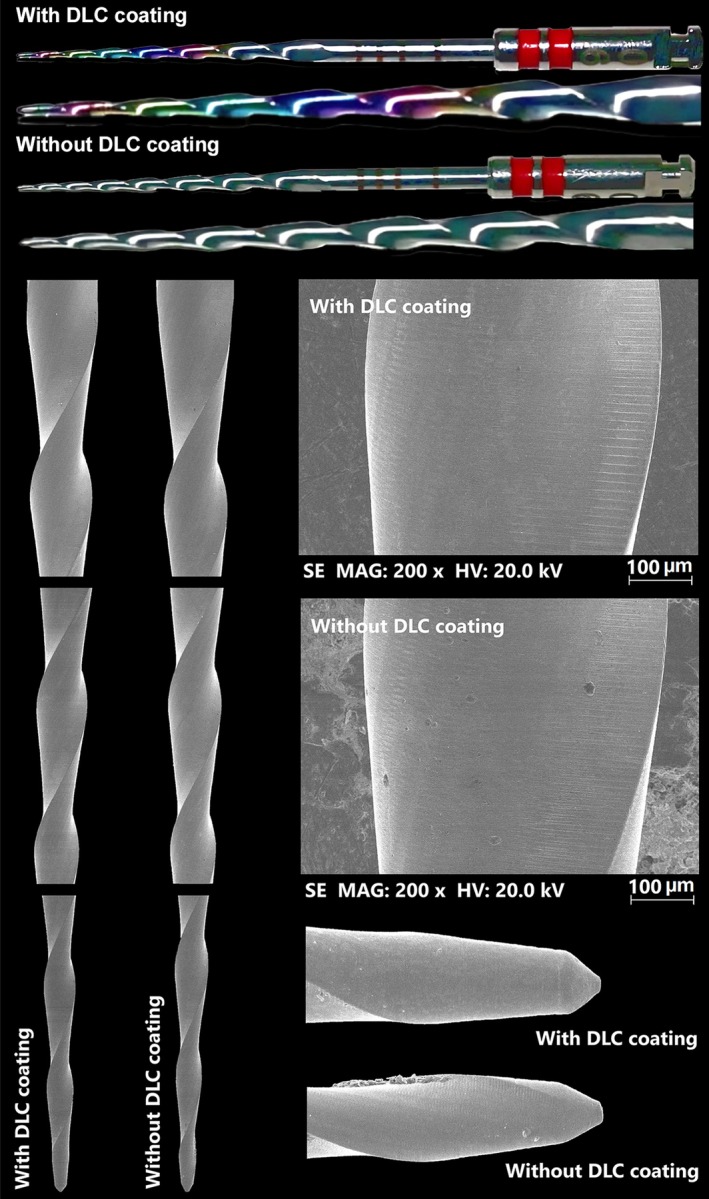
Representative images of the tested instruments. The macro photographs (top) show two identical instruments, with the primary distinction being the multicoloured appearance of the alloy in the DLC‐coated instrument, whereas the non‐coated instrument exhibits a uniform, monochromatic finish. Scanning electron microscopy (SEM) analysis confirmed that both instruments shared a similar geometric design in the blade region (left) and at the tip (bottom right). Additionally, high‐magnification SEM images of the surface finish (middle right) revealed fewer irregularities in the DLC‐coated instrument, indicating a smoother and more refined surface texture compared with the non‐coated counterpart.

The design analysis confirmed that both instruments had consistent geometric designs, with a 15 mm active cutting blade, seven spirals (0.47 spirals per millimetre), and clockwise spiral orientation. SEM inspection showed similar spiral and tip designs, with DLC‐coated instruments exhibiting fewer surface irregularities. The main difference between the groups was the multi‐coloured DLC‐coated instruments, contrasting with the monochromatic non‐DLC instruments (Figure 1).

### Metallurgy

**FIGURE 2:**
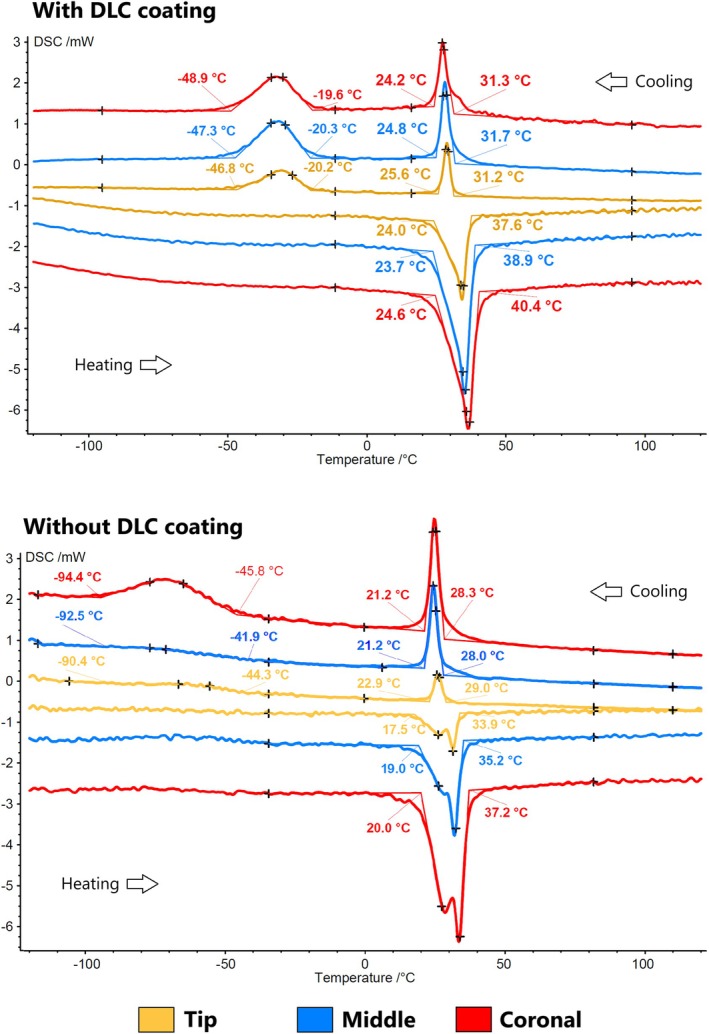
Differential scanning calorimetry (DSC) analysis of the tested instruments. The DLC‐coated instruments (top) exhibited identical transformation temperatures and DSC curves across the three tested fragments obtained from different blade sections (tip, middle, and coronal). A similar pattern was observed in the non‐coated instruments (bottom), indicating consistent thermal behaviour throughout the blade. The only relevant differences were the transition from the R‐phase to the B19’ phase and the presence of a double peak in the heating curve of the non‐coated instruments. Aside from these variations, no significant differences in phase transformation temperatures were detected between the coated and non‐coated instruments.

The three fragments from different sections of the active cutting blade in both DLC‐coated and non‐DLC instruments exhibited identical transformation temperatures during cooling and heating. However, for non‐DLC instruments, distinct peaks were observed during the B19’ to R‐phase and R‐phase to B2 transformations in heating. Relevant differences were found in the transition from the R‐phase to B19’ during heating, which began close to −20°C for DLC‐coated files and −50°C for non‐DLC files. Additionally, DLC‐coated instruments displayed a single peak for the B19’ to B2 transition in the heating curve (martensitic to austenitic state), indicating the superposition of both transformation peaks (B19’ to R‐phase and R‐phase to B2), whilst non‐DLC instruments showed a double peak. Despite these differences, phase transformation temperatures were consistent across both groups, with Rs between 28 and 32°C, Rf between 21 and 25°C during cooling, and Af between 33 and 40°C (Figure 2).

### Mechanical performance

Diamond‐like carbon‐coated instruments demonstrated significantly superior cyclic fatigue resistance (*p* = 0.0028), greater flexibility, as evidenced by a lower bending load (*p* = 0.0294), lower cutting efficiency (*p* < 0.0001), and higher microhardness (*p* = 0.0019) when compared with the instruments without DLC treatment. No significant differences were observed between the two groups in terms of buckling strength (*p* = 0.3569) (Table 1).

**TABLE 1:** Mean (±standard deviation) and median [first and third quartile] results of time to fracture, maximum bending load (in gf), buckling strength (in gf), cutting ability (in gf), and microhardness (HVN) of tested instruments.

	Instrument with DLC	Instrument without DLC	*p* value
Time to fracture	67 ± 13	47 ± 12	*p* = 0.0028
Bending load	331 ± 37	371 ± 37	*p* = 0.0294
Buckling strength	225 ± 26	214 ± 23	*p* = 0.3569
Cutting ability	111 ± 9	84 ± 8	*p* < 0.0001
Microhardness	367 [334–381]	320 [299–338]	*p* = 0.0019

## DISCUSSION

Diamond‐like carbon coatings have been extensively studied in biomedical applications due to their ability to enhance hardness, wear resistance, and chemical stability (Malisz et al., 2023; Penkov et al., 2018; Roy & Lee, 2007). These coatings are applied through a solid‐state ionization process in a vacuum furnace, forming a thin carbon‐based film that modifies the instrument's surface properties. Depending on the specific DLC deposition technique and processing parameters, the resulting coating may exhibit a range of optical characteristics, including different colours or iridescent effects. The present study is the first to comprehensively evaluate the effect of DLC surface treatment on the mechanical properties of rotary NiTi instruments, providing new insights into its influence on cyclic fatigue resistance, buckling resistance, flexibility, cutting efficiency, and microhardness. To accurately assess the impact of DLC treatment, a controlled study design was implemented, comparing instruments identical in all aspects except for the presence of the coating. The design analysis confirmed that the geometric features of instruments with and without DLC coating were similar, with the only noticeable difference being the multi‐coloured appearance of the active blade in the DLC‐coated instruments. This colour variation resulted from the coating process, which also contributed to a smoother surface with fewer irregularities (Figure 1). The colour pattern observed in the RCS Rainbow instruments reflects a standardized DLC coating process, where minor tonal variations arise from optical interference phenomena inherent to thin‐film coatings, without compromising the structural or mechanical integrity of the instruments. Additionally, DSC analysis demonstrated consistent heat treatment along the cutting blade in both groups, with minor variations attributed to the DLC application rather than differences in heat treatment (Figure 2). These modifications did not significantly affect phase transformation temperatures within the clinically relevant range. However, DLC‐coated instruments exhibited significantly greater cyclic fatigue resistance, flexibility, and microhardness compared with their non‐coated counterparts (Table 1), leading to the rejection of the null hypothesis.

The superior fatigue resistance observed in the DLC‐coated instruments (Table 1) suggests that the coating may contribute to a more uniform stress distribution along the instrument, reducing localized stress concentrations that could lead to failure. Additionally, the DLC coating creates a smoother surface finish (Figure 1), which may minimize the risk of crack initiation and propagation by eliminating surface irregularities that act as stress concentrators. This finding is consistent with previous reports (Hinüber et al., 2010; Malisz et al., 2023) indicating that DLC coatings can reduce surface roughness and enhance resistance to microfractures, further supporting their potential role in improving instrument durability. Moreover, the increased surface hardness from DLC coatings may enhance corrosion resistance to endodontic irrigants like sodium hypochlorite, as the carbon‐based layer helps prevent surface degradation and extends the lifespan of NiTi instruments. Whilst promising, this benefit requires further investigation.

Diamond‐like carbon coatings are known for their excellent mechanical properties, including high hardness and elastic modulus, which can enhance the durability and wear resistance of medical instruments (Malisz et al., 2023; Rothammer et al., 2021). However, the flexibility of a material is often inversely related to its hardness and modulus. In the context of DLC coatings, the literature suggests that whilst these coatings improve wear resistance and durability, they may also increase the stiffness of the coated instruments due to their high elastic modulus (Rothammer et al., 2021). The present results demonstrate that DLC‐coated instruments exhibited greater flexibility than their non‐coated counterparts (Table 1). This finding may be attributed to the specific type of DLC coating applied to the NiTi alloy. Literature evidence suggests that whilst standard DLC coatings are generally associated with increased stiffness, specific alterations can improve flexibility. For instance, Yang et al. (2019) demonstrated that the incorporation of immobilized C60 fullerene clusters enhanced elasticity and damping capacity, whilst Leonard et al. (2023) reported that doping DLC films with silicon oxide can reduce the modulus and hardness, potentially increasing flexibility compared with conventional DLC coatings. Given that modifications in the DLC coating procedure can enhance flexibility, it is plausible to assume that the manufacturer of the Rainbow instruments implemented similar adjustments to optimize their mechanical properties. Unfortunately, as this is a proprietary process, we did not have access to detailed information regarding the specific modifications applied.

The cutting efficiency of endodontic instruments is influenced by several key factors, including blade sharpness, surface hardness, and frictional properties (McSpadden, 2007). A sharper blade allows for more precise dentine removal with less applied force, whilst increased surface hardness enhances durability and maintains cutting performance over time. Additionally, the frictional properties of the instrument play a crucial role in reducing resistance during canal preparation, minimizing heat generation, and preventing unnecessary stress on the instrument and surrounding dentine. A recent review highlighted the tribological properties of DLC coatings, such as high hardness and low friction, which are beneficial for applications requiring wear resistance and durability (Malisz et al., 2023). These properties suggest that DLC coatings could indeed enhance the cutting efficiency of medical instruments by reducing wear and maintaining sharpness over time. Furthermore, the study by Xiong et al. (2023) discussed the mechanical behaviours of DLC films, emphasizing their high hardness and wear resistance, which are crucial for maintaining the cutting edge of instruments. According to these authors, the ability of DLC coatings to provide a self‐lubricating surface can also contribute to smoother cutting actions, potentially increasing efficiency. However, in the present study, DLC coating reduced the cutting efficiency of the instrument (Table 1). It is important to recognize that the impact of DLC coatings on cutting efficiency is highly dependent on the specific application and the properties of the substrate material. Whilst DLC coatings are known for their hardness, wear resistance, and low friction, their influence on performance can vary under different conditions. For instance, it has been reported an increase in surface roughness and friction in certain DLC‐coated applications, which, if not properly managed, could negatively affect cutting efficiency (Döring et al., 2019). Additionally, the enhanced flexibility imparted by the DLC coating may alter the mechanical behaviour of the instrument, potentially reducing its cutting effectiveness (McSpadden, 2007). This reduction in cutting efficiency suggests that DLC treatment could present a clinical drawback by increasing the force required for dentine removal. A higher cutting force may, in turn, elevate the mechanical stress exerted on the instrument, which could impact its performance during root canal procedures. However, further research is necessary to determine whether these potential drawbacks significantly affect the instrument's shaping ability in clinical practice. Advanced imaging techniques, such as micro‐computed tomography (micro‐CT), could provide valuable insights into the effects of DLC coatings on root canal preparation.

Interestingly, no significant differences were observed between the coated and non‐coated instruments in terms of buckling resistance (Table 1). Buckling occurs when an instrument is subjected to excessive axial loading, causing it to deform before reaching the point of fracture. This mechanical property is primarily determined by factors, such as core diameter, geometric design, and overall structural rigidity. Since both instrument versions shared an identical design, the lack of significant differences in buckling resistance suggests that DLC treatment does not substantially impact the instrument's macroscopic mechanical behaviour under axial compression. The enhanced surface hardness resulting from the DLC coating might have mitigated the instrument's higher flexibility, thereby possibly influencing the observed similarity in buckling resistance between the coated and non‐coated versions. This finding indicates that whilst DLC coatings enhance certain properties, such as surface hardness and cutting efficiency, they do not compromise the structural integrity of the instrument when subjected to compressive forces.

A primary limitation of this study is that only a subset of mechanical properties was evaluated, focusing specifically on those claimed by the manufacturer to be enhanced by DLC treatment. This selective approach was intended to assess the most relevant mechanical improvements. Future studies should expand the scope of mechanical testing, to further elucidate the impact of DLC coatings on NiTi instruments. Moreover, whilst mechanical tests provide crucial insights into instrument performance, they capture only a limited aspect of its behaviour. To gain a more comprehensive understanding of how DLC treatment influences root canal instrumentation, future studies should incorporate the micro‐CT analytical tool. Additional studies should also assess whether the wear‐reducing effects of DLC coatings vary with the type of thermal treatment applied to the NiTi alloy or remain consistent across different metallurgical conditions. This could clarify whether DLC technology provides universal benefits regardless of the alloy's phase composition or mechanical properties. Despite these limitations, this study presents several strengths. By employing a multimethod approach, the research provided a robust and data‐driven evaluation of the effects of DLC coatings on NiTi instruments. Moreover, the controlled study design—where the only variable was the presence or absence of DLC coating—ensured that all observed differences were directly attributable to the surface treatment, eliminating confounding factors related to instrument geometry, metallurgical properties, or manufacturing inconsistencies. This methodological rigour enhances the validity of the results and provides a solid foundation for future research exploring the clinical implications of DLC‐coated NiTi instruments.

The findings of this study provide evidence that DLC coatings positively impact the mechanical properties of NiTi rotary instruments, enhancing their cyclic fatigue resistance and flexibility whilst maintaining structural integrity. The increased fatigue resistance suggests improved longevity, reducing the risk of instrument fracture during clinical use. Greater flexibility enhances adaptability to complex canal anatomies, potentially minimizing procedural errors. Collectively, these advantages indicate that DLC‐treated instruments could offer superior durability and performance, ultimately contributing to safer and more efficient root canal preparation. However, further clinical investigations are warranted to fully assess the long‐term benefits and potential implications of DLC coatings in endodontic practice.

## CONCLUSION

Diamond‐like carbon surface treatment significantly enhanced cyclic fatigue resistance, flexibility, and microhardness of NiTi rotary instruments without compromising their structural integrity.

## AUTHOR CONTRIBUTIONS

**Emmanuel J. N. L. Silva:** conceptualization, analysis, experimental procedures, writing, review, and editing (lead). **Tereza Vitória Mauri Lorenzoni:** experimental procedures. **Mylena do Rosário Pereira:** experimental procedures. **Victor T. L. Vieira:** experimental procedures, writing. **Jorge N. R. Martins:** conceptualization, analysis, experimental procedures, writing, review, and editing (lead). **Francisco Manuel Braz Ferndandes:** experimental procedures. **Marco A. Versiani:** conceptualization, analysis, experimental procedures, writing, review, and editing (lead).

## FUNDING INFORMATION

This study was partially funded by FAPERJ and CNPq.

## CONFLICT OF INTEREST STATEMENT

The authors declare that they have no competing interests with regard to this paper.

## ETHICS STATEMENT

Not applicable.

## Supporting information


Figure S1.

## Data Availability

Data available on request from the authors.
